# Marine Mammal *Brucella* Reference Strains Are Attenuated in a BALB/c Mouse Model

**DOI:** 10.1371/journal.pone.0150432

**Published:** 2016-03-09

**Authors:** Ingebjørg H. Nymo, Maykel A. Arias, Julián Pardo, María Pilar Álvarez, Ana Alcaraz, Jacques Godfroid, María Pilar Jiménez de Bagüés

**Affiliations:** 1 Arctic Infection Biology, The Faculty of Biosciences, Fisheries and Economics, University of Tromsø (UiT)–The Arctic University of Norway, Tromsø, Norway; 2 Unidad de Tecnología en Producción y Sanidad Animal, Centro de Investigación y Tecnología Agroalimentaria (CITA), Instituto Agroalimentario de Aragón – IA2 (CITA-Universidad de Zaragoza), Zaragoza, Spain; 3 Cell Immunity in Cancer, Inflammation and Infection Group, Department of Biochemistry and Molecular and Cell Biology, Biomedical Research Centre of Aragon (CIBA), IIS Aragón/University of Zaragoza, Zaragoza, Spain; 4 Laboratorio Agroalimentario, Gobierno de Aragón, Zaragoza, Spain; 5 College of Veterinary Medicine, Western University of Health Sciences, Pomona, California, United States of America; Purdue University, UNITED STATES

## Abstract

Brucellosis is a zoonosis of worldwide distribution with numerous animal host species. Since the novel isolation of *Brucella* spp. from marine mammals in 1994 the bacteria have been isolated from various marine mammal hosts. The marine mammal reference strains *Brucella pinnipedialis* 12890 (harbour seal, *Phoca vitulina*) and *Brucella ceti* 12891 (harbour porpoise, *Phocoena phocoena*) were included in genus *Brucella* in 2007, however, their pathogenicity in the mouse model is pending. Herein this is evaluated in BALB/c mice with *Brucella suis* 1330 as a control. Both marine mammal strains were attenuated, however, *B*. *ceti* was present at higher levels than *B*. *pinnipedialis* in blood, spleen and liver throughout the infection, in addition *B*. *suis* and *B*. *ceti* were isolated from brains and faeces at times with high levels of bacteraemia. In *B*. *suis*-infected mice serum cytokines peaked at day 7. In *B*. *pinnipedialis*-infected mice, levels were similar, but peaked predominantly at day 3 and an earlier peak in spleen weight likewise implied an earlier response. The inflammatory response induced pathology in the spleen and liver. In *B*. *ceti*-infected mice, most serum cytokine levels were comparable to those in uninfected mice, consistent with a limited inflammatory response, which also was indicated by restricted spleen and liver pathology. Specific immune responses against all three strains were detected *in vitro* after stimulation of splenocytes from infected mice with the homologous heat-killed brucellae. Antibody responses *in vivo* were also induced by the three brucellae. The immunological pattern of *B*. *ceti* in combination with persistence in organs and limited pathology has heretofore not been described for other brucellae. These two marine mammal wildtype strains show an attenuated pattern in BALB/c mice only previously described for *Brucella neotomea*.

## Introduction

*Brucella* spp. causes brucellosis, a zoonosis of almost worldwide distribution [1]. *Brucella*-infection is characterized by bacterial persistence in the reticuloendothelial system and replication of the organism in the reproductive system in primary hosts is associated with abortion and sterility [2]. Genus *Brucella* consists of eleven species, the six classical species: *Brucella melitensis*, *Brucella abortus*, *Brucella suis*, *Brucella ovis*, *Brucella neotomae* and *Brucella canis* [1] and the more recently added species: *Brucella pinnipedialis*, *Brucella ceti* [3], *Brucella microti* [4], *Brucella inopinata* [5] and *Brucella papionis* [6].

*Brucella* spp. were first reported from marine mammals in 1994 [7] and validly published as two novel species for inclusion in the genus *Brucella* in 2007 as *Brucella pinnipedialis* and *Brucella ceti* with pinnipeds and cetaceans as preferred hosts respectively [8]. The type strains for these species are *B*. *pinnipedialis* NCTC 12890 (hereafter entitled *B*. *pinnipedialis* 12890), isolated from the spleen of a harbour seal (*Phoca vitulina*) and *Brucella ceti* NCTC 12891 (hereafter entitled *B*. *ceti* 12891), isolated from a skin lesion on a harbour porpoise (*Phocoena phocoena*) [3]. Marine mammal brucellae have later been isolated from several pinniped and cetacean species, yet reports on the pathological findings in association to *B*. *pinnipedialis*-infection in true seals (*Phocidae*) are virtually absent, and the pathological findings in association to infection with *B*. *ceti* in harbour porpoises are scarce. In contrast, *B*. *ceti* has been isolated from various lesions in dolphins, in particular from the central nervous system associated with neurological pathology and live stranding, but also from uterus, testes, aborted foetuses, vaginal secretions, milk and mammary glands [9–12]. Additionally, an unusual genotype (sequence type 27, ST27) [13] only isolated thus far from bottlenose dolphins (*Tursiops truncatus*)[14] and a California sea lion (*Zalophus californianus*) [15] has been shown to multiply in human macrophages to the same degree as *B*. *suis* 1330 [16] and has also caused naturally acquired infections in humans [17, 18]. The ST27 isolates share a unique variable number of tandem repeats (VNTR) profile and restriction fragment length polymorphism (RFLP) analysis of outer membrane protein 2 (*omp2*)–encoding genes also showed an identical genotype among the isolates [13].

*In vitro* work has shown that *B*. *pinnipedialis* 12890 did not multiply in murine, human or hooded seal (*Cystophora cristata*) macrophages, or in a human epithelial cell line [19, 20] and *B*. *ceti* 12891 did not multiply in human [16] or hooded seal macrophages [20]. Other work displayed deviating results by showing that *B*. *pinnipedialis* 12890 was able to multiply in human macrophages to the same extent as the virulent strains *B*. *suis* 1330 and *B*. *melitensis* 16M [16].

Experimental infection in piglets (*Sus scrofa domesticus*) with a marine mammal brucellae human isolate 02/611 [18], belonging to ST27 [13], yielded no pathology [21], while abortion has been induced in cattle (*Bos taurus*) after infection with a *B*. *pinnipedialis* Pacific harbour seal (*Phoca vitulina richardsi*) strain [22]. Experimental infection of pregnant sheep (*Ovis aries*) [23] with a *B*. *pinnipedialis* harbour seal strain and *B*. *ceti* harbour porpoise and common dolphin (*Delphinus delphis*) strains [7] resulted in limited pathology, while infection in guinea pigs (*Cavia porcellus*) with the same strains resulted in splenomegaly and high antibody titres [23]. Another guinea pig study showed that *B*. *ceti* from a bottlenose dolphin foetus was less virulent than the terrestrial strains *B*. *abortus* S19, *B*. *abortus* B3196, *B*. *melitensis* 16M and *B*. *melitensis* 63/9 [14]. A hooded seal *B*. *pinnipedialis* strain showed reduced pathogenicity in BALB/c mice (*Mus musculus*) as compared to *B*. *suis* 1330 [24].

Heretofore, *in vivo* experimental infections with marine mammal brucellae have been performed with various strains in different animal species, under various conditions, and with diverse and non-conclusive results [14, 21–23]. A murine model of infection for *Brucella* spp., however, is well established and much of the current understanding of the pathogenesis and immunity of animal brucellosis has arisen from studies in mice [25–27]. The Subcommittee on the Taxonomy of the Genus *Brucella* has in the minimal standards for recognition of an organism as a member of the genus stated that pathogenicity in mice should be investigated [8]. This work is lacking for the marine mammal reference strains. The aim of the present study was to evaluate the pathogenicity of *B*. *pinnipedialis* 12890 and *B*. *ceti* 12891 in a BALB/c mouse model by characterizing the evolution of the infection, histopathology and immunological parameters.

## Materials and Methods

### Ethics statement

Three mouse experiments were conducted in strict accordance with the guidelines from the Federation of Laboratory Animal Science Associations and were approved by the Agrifood Research and Technology Centre of Aragon (CITA) ethical animal experiment committee. No mice died during the experiments. The main (I) murine infection (comprising bacteriology, histopathology, serum antibody isotypes and serum cytokines) lasted for 12 weeks and is described under “Murine infections”, section one (experiment number: I118/2010/1). A supplementary murine infection (II), for the detection of early serum cytokines, lasting for three days, is described under “Murine infections”, section two (experiment number: I124/2014/2). A third mouse experimental infection (III), lasting for 7 days, was performed to do an *in vitro* splenocyte experiment for the detection of supernatant cytokines after stimulation *in vitro* with heat-killed (HK) brucellae. This is described under “*In vitro* splenocyte cytokine responses following antigen stimulation” (experiment number: I125/2014/12).

### Bacterial strains

The strains studied were the marine mammal reference strains *B*. *pinnipedialis* sp. nov. (NCTC 12890^T^, BCCN 94-73^T^) and *B*. *ceti* sp. nov. (NCTC 12891^T^, BCCN 94-74^T^) [3] (kindly provided by Dr. Cloeckaert and Dr. Zygmunt, The INRA Val de Loire Centre, Nouzilly, France). *Brucella suis* biovar 1 strain 1330 (NCTC 10316, ATCC 23444, from now on entitled *B*. *suis* 1330) was included as a classical *Brucella* control. The marine mammal strains were grown on blood agar base N°2 (BAB) (Oxoid, Basingstoke, UK) with 5% newborn calf serum (NCS) (Pan-Biotech GmbH, Aidenbach, Germany) and *B*. *suis* 1330 was grown on BAB, if otherwise is not specified. All strains were incubated aerobically at 37°C (*B*. *pinnipedialis* 12890 in a 5% CO_2_ enriched atmosphere). The expression of smooth surface antigens was verified by crystal violet staining [28]. The identity of the bacteria grown from organ samples was verified by polymerase chain reaction (PCR), as previously described [24] (results not shown).

### Murine experiments

In the main murine infection, lasting for 12 weeks, eight week old female BALB/c mice (Charles River Laboratories, L'Arbresle Cedex, France) were injected intraperitoneally (ip) with 100 μl sterile phosphate buffered saline (sPBS) holding 10^5^ colony forming units (CFU) of *B*. *pinnipedialis* 12890, *B*. *ceti* 12891 or *B*. *suis* 1330. To prepare the infective solution the strains were grown for 48 (marine mammal strains) or 24 hrs (*B*. *suis* 1330) and thereafter diluted in sPBS. Uninfected control mice received 100 μl sPBS ip. Four or five mice were euthanized by CO_2_ asphyxiation per lot at day 3, 7, 14, 21, 35, 56 and 84 post infection (pi) (day 56 and 84; only *B*. *ceti* 12891 and *B*. *suis* 1330). Spleen, liver, brain and faecal samples were collected aseptically and weighted. Blood was sampled by cardiac puncture. A portion of the blood was used for bacteriology while the rest was centrifuged (3,000 rpm, 15 min, RT°) to obtain serum. Sera were passed through 0.2 μm Acrodisc Syringe Filters with Supor Membrane (Pall Corporation, NY, USA) and stored at -80°C.

As mice infected with *B*. *ceti* 12891 produced a limited serum cytokine response on day 3, 7 and 14 pi (see “Results”, “Serum cytokines”) we performed an additional murine infection, identical to the main murine infection described above, but lasting only for three days, for the detection of early (day 1, 2 and 3 pi) serum cytokines. Eight week old female BALB/c mice (Janvier Labs, Le Genest-Saint-Isle, France) were injected ip with 10^5^ CFU of *B*. *pinnipedialis* 12890, *B*. *ceti* 12891 or *B*. *suis* 1330. Uninfected control mice were treated as described above. Four or five mice were euthanized per lot at day 1, 2 and 3 pi and spleen and serum samples were collected as described above. From these mice spleen bacterial loads (to verify that the level of infection was comparable to that in the main investigation) and serum cytokine levels were investigated.

### Bacteriology

Weighed portions of spleen, liver and brain from mice infected with *B*. *pinnipedialis* 12890, *B*. *ceti* 12891 or *B*. *suis* 1330 in the main investigation were homogenized, serially diluted and plated (marine mammal strains; BAB + 5% NCS/*B*. *suis* 1330; BAB) for evaluation of the number of CFU. Faecal samples were treated the same way, but were plated on modified Farrell medium (one vial of *Brucella* selective supplement (Oxoid) per BAB litre + 5% NCS). Blood was plated for the evaluation of CFU as described above. The numbers of bacterial counts were logarithmic transformed (except for the faeces results) and expressed as mean + one standard deviation (SD). When no colonies were detected a limit of detection of at least one colony was assigned for the log conversion.

### Histopathology

Mice infected with *B*. *pinnipedialis* 12890, *B*. *ceti* 12891 or *B*. *suis* 1330 in the main investigation and uninfected control mice, were examined at sacrifice for gross pathology. Samples of spleen, liver and brain were kept in 4% formaldehyde in sPBS until processing. Samples examined from all animals included three sections from the right and left hepatic lobes as well as the right middle lobe, including the gallbladder and one section of spleen. Three transverse sections of the brain, including portions of cortex, *corpus callosum*, hippocampus, pons and cerebellum, were examined. Fixed tissues were embedded in paraffin, cut in 4 μm sections and stained with haematoxylin and eosin for histopathological examination. Sections were analysed blinded for light microscopy analysis, and the main lesions were counted and described in all stained sections by a Diplomat of the American College of Veterinary Pathologists.

### Serum antibody isotypes

Serum samples from mice infected with *B*. *pinnipedialis* 12890, *B*. *ceti* 12891 or *B*. *suis* 1330 in the main investigation were tested for the levels of antibody isotypes, immunoglobulin (Ig) M, IgG1, IgG2a and IgG3, with enzyme-linked immunosorbent assays (ELISAs) with *B*. *abortus* lipopolysaccharide as antigen, as previously described [24]. The results are expressed as mean optical density (od) + one SD.

### Serum cytokines

Serum levels of granulocyte-macrophage colony-stimulating factor (GM-CSF), interleukin (IL)-1β, tumor necrosis factor (TNF)-α, interferon (IFN)-γ, IL-2, IL-12 (p40/p70), IL-4, IL-5, IL-6 and IL-10 were determined in mice infected with *B*. *pinnipedialis* 12890, *B*. *ceti* 12891 or *B*. *suis* 1330 utilizing the Cytokine Mouse 10-Plex Panel (Invitrogen) on the Luminex 100^™^ analyzer (Luminex Corporation, Austin, TX, USA). Sera from day 3, 7 and 14 pi were analysed during the main murine infection (“Murine infections”, section one). Sera from day 1, 2 and 3 pi were analysed during an additional murine infection performed for the detection of early serum cytokines (“Murine infections” section two), with the same Cytokine Mouse 10-Plex Panel. Serum cytokines and spleen bacterial load on day 3 pi were analysed during both experiments to ensure coherence. Serum cytokine levels are presented as mean levels in pg/ml + one standard error of the mean (SEM).

### *In vitro* splenocyte cytokine responses following antigen stimulation

In a separate, third, experiment, BALB/c mice were infected with *B*. *pinnipedalis* 12890, *B*. *ceti* 12891 or *B*. *suis* 1330, as described under “Murine infections”. On day 7 pi a suspension of splenocytes from infected and uninfected mice were obtained as formerly described [29] and cultured in 24-well sterile plates (2 x 10^6^ cells/well). Splenocytes from mice infected with *B*. *pinnipedalis* 12890, *B*. *ceti* 12891 or *B*. *suis* 1330 were subsequently either stimulated with the homologous HK *B*. *pinnipedalis* 12890, HK *B*. *ceti* 12891 or HK *B*. *suis* 1330, at a multiplicity of infection of 100, or left unstimulated. Splenocytes from uninfected mice were stimulated with the three strains of HK brucellae, or left unstimulated. Additionally, splenocytes from infected and uninfected mice were stimulated with LPS from *Escherichia coli* serotype O55:B5 (Sigma Aldrich, Madrid, Spain) 100ng/ml. Spleen cells were incubated at 37°C in a 5% CO_2_ enriched atmosphere for 72 h as described [29], after which supernatants were harvested, centrifuged (1,200 RPM, 5 min, RT°), passed through 0.2 μm Acrodisc Syringe Filters with Supor Membrane (Pall Corporation), and used for measuring IFN-γ, TNF-α and IL-6 levels with the Ready-Set-Go! ELISA kits from eBioscience (San Diego, CA, USA). The experiment was repeated twice and the results are presented together. Results are presented as mean level of cytokines in pg/ml + one SEM.

### Statistics

All comparisons were made by an unpaired two-tailed Student t-test. P values < 0.05 were considered significant.

## Results

### Bacteriology and organ weights

**Fig 1:**
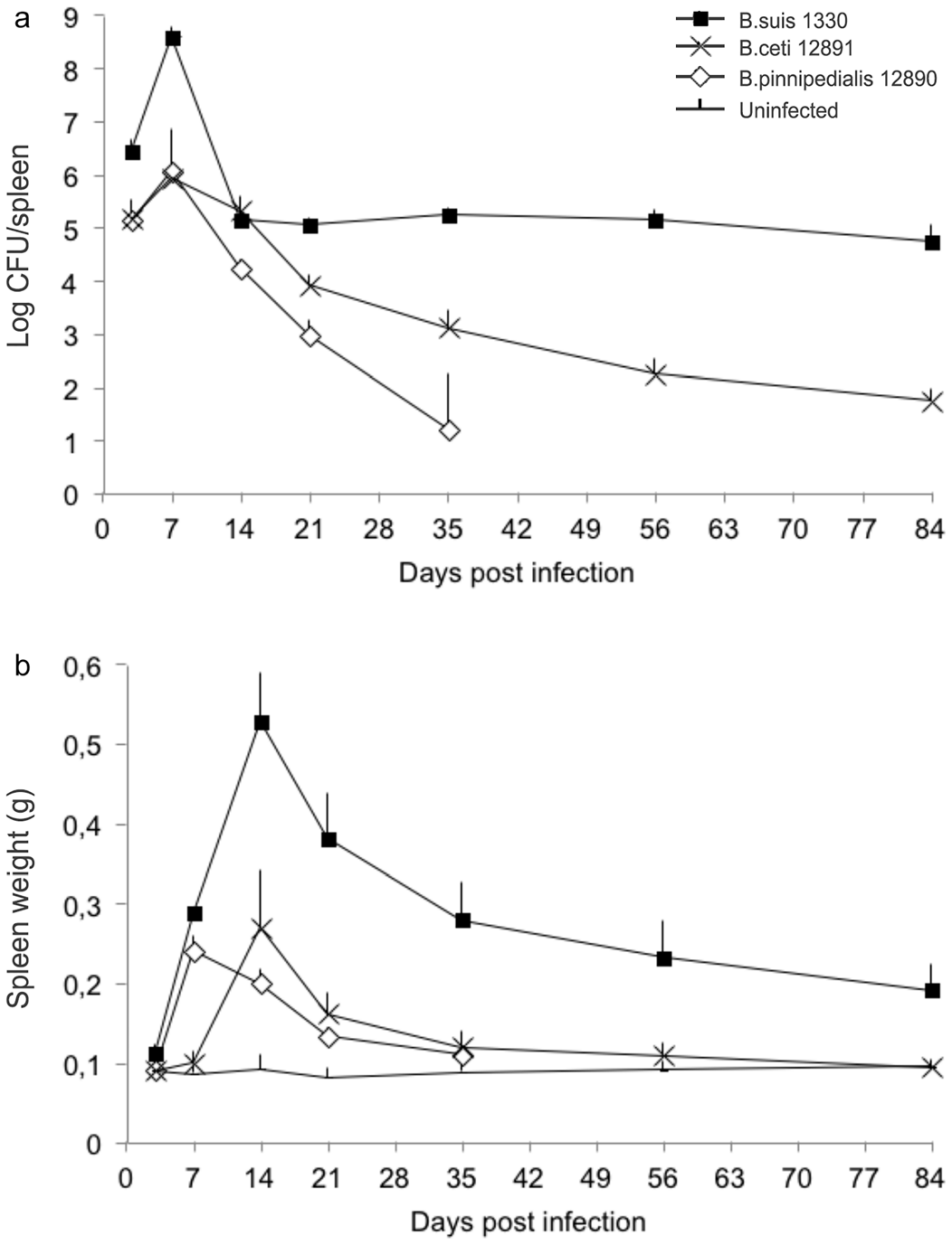
Spleen bacterial counts and organ weights. Bacterial counts for B. *pinnipedialis* 12890 (open diamonds), *B*. *ceti* 12891 (crosses) and *B*. *suis* 1330 (black squares) in spleens (a) of BALB/c mice after intraperitoneal (ip) inoculation of 10^5^ colony forming units (CFU) of bacteria. Uninfected control mice received sterile phosphate buffered saline ip (black lines). Four or five mice were euthanized per lot at day 3, 7, 14, 21, 35, 56 and 84 post infection (day 56 and 84; only *B*. *ceti* 12891 and *B*. *suis* 1330). The number of viable bacteria was determined and the numbers of bacterial counts were logarithmic transformed. Spleen weights (b) were determined in parallel. Results are expressed as mean + one standard deviation. Whether CFU numbers differed significantly between mice infected with *B*. *suis* 1330, and *B*. *ceti* 12891 or *B*. *pinnipedialis* 12890, respectively, at the different times pi, are presented in Table 1. Whether spleen weights of the infected mice were significantly different from those of the uninfected control mice, at the different times pi, is presented in S1 table.

The spleen and liver bacterial counts (Fig 1a and S1 Fig) and weights (Fig 1b and S1 Fig) in mice infected with *B*. *suis* 1330 were similar to those described in the literature [30]. The numbers of CFU in the spleens of mice infected with the marine mammal brucellae reference strains were significantly lower than those found in mice infected with *B*. *suis* 1330 at all times pi (p < 0.001), except for mice infected with *B*. *ceti* 12891 at day 14, where there was no significant difference (Table 1). Mice infected with *B*. *pinnipedialis* 12890 showed an increase in spleen CFU between day 3 (5.13 log CFU ± 0.37) and 7 (6.05 log CFU ± 0.17) and a subsequent decline reaching 1.22 log CFU (± 1.02) at day 35. The spleen bacterial counts in mice infected with *B*. *ceti* 12891 followed the same pattern as that of mice infected with *B*. *pinnipedialis* 12890 at day 3 and 7, while at day 14 (p < 0.001), 21 (p < 0.001) and 35 (p < 0.01) the spleen CFU numbers were significantly higher in mice infected with *B*. *ceti* 12891, as compared to those infected with *B*. *pinnipedialis* 12890 (Fig 1a). The spleen weight in mice infected with *B*. *pinnipedialis* 12890 peaked at day 7 and at day 35 the spleen weights did not differ significantly from those found in uninfected control mice. The spleen weight of mice infected with *B*. *ceti* 12891 peaked at day 14 and at day 35 the weights did not differ significantly from those found in uninfected control mice (Fig 1b and S1 Table). In the liver we observed the same patterns for bacterial counts (S1 Fig and Table 1) and weights (S1 Fig and S1 Table) as in the spleen, confirming the attenuation of the marine mammal reference strains in the BALB/c mouse model.

**Table 1:** Level of statistical difference when comparing numbers of colony forming units in BALB/mice infected with *Brucella suis* 1330 to those infected with the marine mammal reference strains.

Day		3	7	14	21	35	56	84
**Spleen CFU**	*Brucella pinnipedialis* 12890	***	***	***	***	***	X	X
	*Brucella ceti* 12891	***	***	ns	***	***	***	***
**Liver CFU**	*Brucella pinnipedialis* 12890	***	**	***	***	***	X	X
	*Brucella ceti* 12891	***	ns	ns	***	**	**	***
**Blood CFU**	*Brucella pinnipedialis* 12890	ns	**	X	X	X	X	X
	*Brucella ceti* 12891	ns	***	**	**	ns	X	X

Level of statistical difference between the number of colony forming units (CFU) in spleen, liver and blood of BALB/c mice after intraperitoneal (ip) inoculation of 10^5^ colony forming units (CFU) of *B*. *suis* 1330, B. *pinnipedialis* 12890 or *B*. *ceti* 12891. Four or five mice were euthanized per lot at day 3, 7, 14, 21, 35, 56 and 84 post infection (day 56 and 84; only *B*. *ceti* 12891 and *B*. *suis* 1330). Mice infected with *B*. *ceti* 12891 or *B*. *pinnipedialis* are compared to mice infected with *B*. *suis* 1330 and *** = p < 0.001, ** = p < 0.01, * = p < 0.05, ns = not significant, X = not available.

The bacteraemia induced by *B*. *suis* 1330 was more pronounced and longer lasting than that induced by the marine mammal reference strains (Table 1 and S2 Fig). The log number of CFU/ml blood for mice infected with *B*. *pinnipedialis* 12890 peaked at day 7 (1.69 log CFU/ml blood ± 1.36) and mice infected with this strain showed no signs of bacteraemia thereafter. Mice infected with *B*. *ceti* 12891 showed two peaks, at day 3 (1.50 log CFU/ml blood ± 0.88) and at day 14 (1.10 log CFU/ml blood ± 1.51) and displayed bacteraemia until at least day 35.

Brucellae were isolated from the brains of mice infected with *B*. *suis* 1330 at day 3 (0.39 log CFU/g brain ± 0.87) and 7 (1.60 log CFU/g brain ± 1.03) and from mice infected with *B*. *ceti* 12891 at day 14 (1.61 log CFU/g brain ± 1.01). Brucellae were also isolated from faecal samples of mice infected with *B*. *suis* 1330 from day 3 to 35 and from mice infected with *B*. *ceti* 12891 at day 14 (S2 Fig). *Brucella pinnipedialis* 12890 was isolated from neither brain nor faeces. There were no significant differences in the log number of CFU/g of brain or faeces between the mice infected with *B*. *suis* 1330 and *B*. *ceti* 12891.

### Histopathology

Pathological findings in the spleen and liver of BALB/c mice infected with *B*. *pinnipedialis* 12901, *B*. *ceti* 12891 or *B*. *suis* 1330 are summarized in Table 2 (day 3–84 pi) and pictures of spleen and liver histopathology at day 14 are presented in S3 Fig. Except for the enlargement of spleen and liver, no gross pathology was observed.

**Table 2:** Histopathological findings in spleen and liver.

Day	*B*. *suis* 1330		*B*. *pinnipedialis* 12890		*B*. *ceti* 12891	
	Spleen	Liver	Spleen	Liver	Spleen	Liver
3	+/- (2/2)	++/- (3/1)	-	+/- (2/3)	-	-
7	*	++	*	++	+	-
14	*	+++	+	++	+	+
21	++	+++	+	+/- (2/3)	+/- (2/3)	+/- (2/3)
35	++	++/- (4/1)	-	-	-	-
56	+	++	X	X	-	-
84	+ (2/3)	+/- (3/2)	X	X	-	-

Spleen and liver pathology in BALB/c mice after intraperitoneal (ip) inoculation of 10^5^ colony forming units (CFU) of B. *pinnipedialis* 12890, *B*. *ceti* 12891 or *B*. *suis* 1330. Uninfected control mice received sterile phosphate buffered saline ip. Four or five mice were euthanized per lot at day 3, 7, 14, 21, 35, 56 and 84 post infection (day 56 and 84; only *B*. *ceti* 12891 and *B*. *suis* 1330). The pathological changes in spleen were graded as follows;—: no pathological changes, *: expansion of the red pulp and lymphoid depletion, +: follicular hyperplasia, and ++: splenomegaly with expanded red and white pulp and granulomas. The pathological changes in liver were graded as follows;—: no pathological changes, +: livers with few inflammatory nodules without necrosis, ++: livers containing inflammatory nodules with up to 10 cells present, +++: livers with large inflammatory nodules, with or without necrosis. When only some of the animals had lesions this is indicated by showing one or more + and a—; additionally, the number of animals with and without lesions, respectively, are shown in brackets. X = not avaliable.

Splenic lesions were most pronounced in mice infected with the virulent *B*. *suis* 1330. They were characterized by splenomegaly with expansion of the red pulp and diminished white pulp (follicular lymphoid depletion) followed by a marked follicular hyperplasia (reactive nodules) mixed with granulomas after day 14. Mice infected with the marine mammal reference strains showed less severe splenic pathology than those infected with *B*. *suis* 1330, with mainly reactive lymphoid follicles and very few small granulomas. Mice infected with *B*. *pinnipedialis* 12890 displayed lesions in all mice at day 7, 14 and 21, with expansion of the red pulp and lymphoid depletion at day 7 and follicular hyperplasia at day 14 and 21, while at day 35, no pathology could be detected. The pathological changes in the spleens of mice infected with *B*. *ceti* 12891 were less severe than in mice infected with *B*. *pinnipedialis* 12890. The *B*. *ceti* 12891-infected mice displayed follicular hyperplasia in all mice at day 7 and 14, in 40% of the mice at day 21 and no pathology at day 35.

Hepatic granulomas were consistently found in all mice with lesions, irrespective of the strain of *Brucella* that they were exposed to (Table 2 and S3 Fig). Characteristic variably sized inflammatory nodules containing macrophages in place of hepatocytes were found in all affected animals. The strain causing the most severe hepatic pathology was *B*. *suis* 1330. A majority of the mice (75%) infected with *B*. *suis* 1330 showed pathological changes starting at day 3. Granulomatous lesions were found in 60% of the mice until at least day 84. The lesions at day 35 and 56 showed necrosis associated to the inflammatory nodules, but necrosis was not observed at day 84. Mice infected with *B*. *pinnipedialis* 12890 showed less severe liver lesions than those infected with *B*. *suis* 1330 at day 3 and only 40% of the mice were affected. At day 7 and 14 all mice infected with *B*. *pinnipedialis* 12890 showed inflammatory nodules, however, by day 35 there was no liver pathology present. Mice infected with *B*. *ceti* 12891 exhibited only minimal pathologic changes at day 14 (100%) and 21 (40%) and the lesions were resolved by day 35. These changes included small inflammatory nodules with no necrosis.

Brains of all infected animals were examined and no pathological changes were detected. Liver, spleen and brain tissues from uninfected control mice were also examined and no pathological lesions were observed.

### Serum antibody isotypes

**Fig 2:**
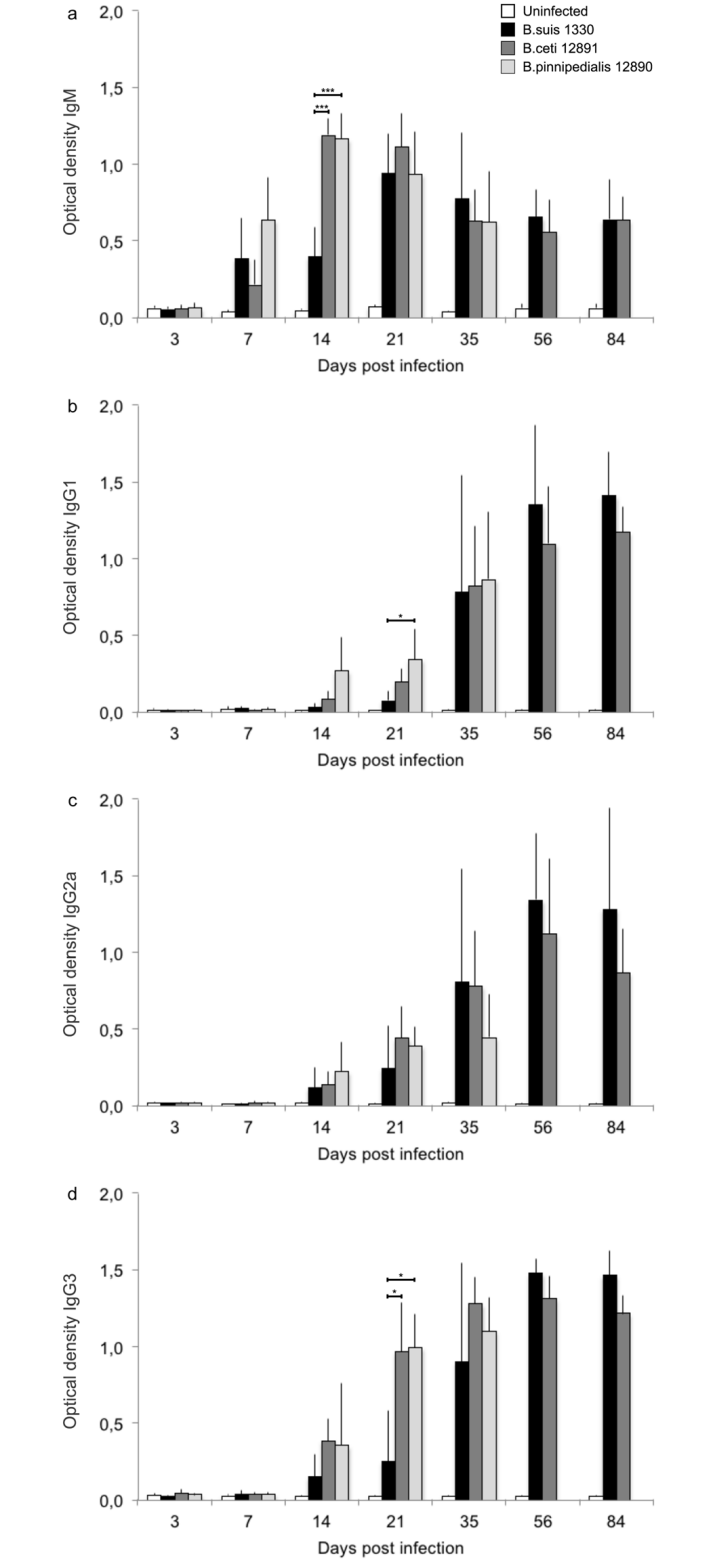
Serum antibody isotypes. Serum antibody isotype patterns for immunoglobulin (Ig) M (a), IgG1 (b), IgG2a (c) and IgG3 (d) in BALB/c mice after intraperitoneal (ip) inoculation of 10^5^ colony forming units (CFU) of *B*. *suis* 1330 (black), B. *pinnipedialis* 12890 (light grey), *B*. *ceti* 12891 (dark grey) or *B*. *pinnipedialis* 12890 (light grey). Uninfected mice received sterile phosphate buffered saline ip (white). Four or five mice were euthanized per lot at day 3, 7, 14, 21, 35, 56 and 84 post infection (day 56 and 84; only *B*. *ceti* 12891 and *B*. *suis* 1330). Data are presented as mean optical density + one standard deviation. The marine mammal reference strains were compared to *B*. *suis* 1330 and *** = p < 0.001, ** = p < 0.01, * = p < 0.05.

There was some variation in the od levels of individual mice, leading to certain sizeable SDs (Fig 2). The mean od levels of the uninfected mice were below 0.072 at all times for all antibody isotypes. The IgM (Fig 2a), IgG1 (Fig 2b), IgG2a (Fig 2c) and IgG3 (Fig 2d) patterns in mice infected with *B*. *suis* 1330 were as described previously for virulent brucellae [24, 26, 31]. Mice infected with *B*. *pinnipedialis* 12890 and *B*. *ceti* 12891 produced patterns similar to those found for *B*. *suis* 1330, however, mice infected with *B*. *pinnipedialis* 12890 and *B*. *ceti* 12891 produced a significantly higher IgM response at day 14 (p < 0.001 and p < 0.01) and IgG3 response at day 21 (p < 0.05) than those infected with *B*. *suis* 1330. At day 21, mice infected with *B*. *pinnipedialis* 12890 produced a significantly higher IgG1 response than those infected with *B*. *suis* 1330 (p < 0.05). There were no significant differences in the IgG2a od values between mice infected with *B*. *suis* 1330 and the marine mammal reference strains. None of the isotype od values were significantly different between the mice infected with the two marine mammal reference strains.

### Serum cytokines

**Fig 3:**
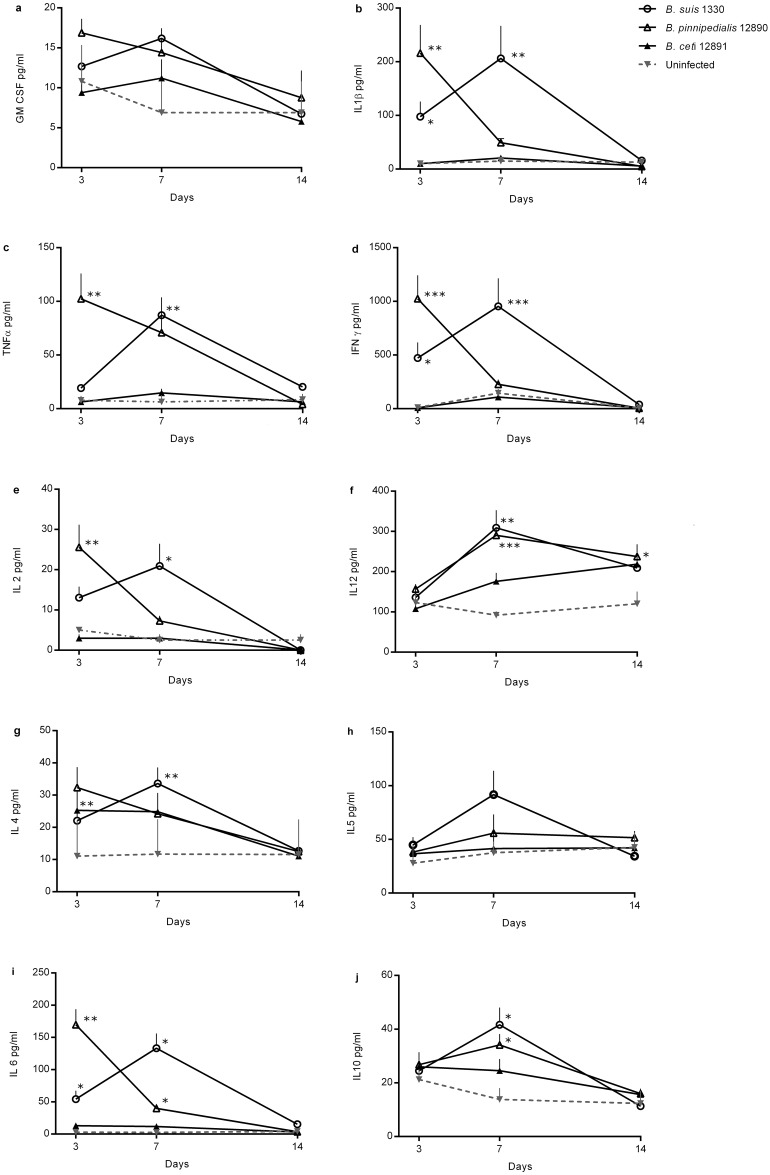
Serum cytokines. Serum levels of granulocyte-macrophage colony-stimulating factor (GM-CSF) (a), interleukin (IL)-1β (b), tumor necrosis factor (TNF)-α (c), interferon (IFN)-γ (d), IL-2 (e), IL-12 (p40/p70) (f), IL-4 (g), IL-5 (h), IL-6 (i) and IL-10 (j) in BALB/c mice, after intraperitoneal (ip) inoculation of 10^5^ colony forming units (CFU) of *B*. *pinnipedialis* 12890, *B*. *ceti* 12891 or *B*. *suis* 1330. Uninfected mice received sterile phosphate buffered saline ip. Four or five mice were euthanized per lot at day 3, 7 and 14 post infection. Results are expressed as mean serum cytokine levels in pg/ml + one standard error of the mean. The marine mammal reference strains were compared to *B*. *suis* 1330 and *** = p < 0.001, ** = p < 0.01, * = p < 0.05.

Sera from day 3, 7 and 14 pi were analysed during the main murine infection (“Murine infections”, section one) (Fig 3), while sera from day 1, 2 and 3 pi were analysed during an additional murine infection performed solely for the detection of early serum cytokines (“Murine infections” section two). Serum cytokines and spleen bacterial load on day 3 pi were analysed during both experiments and neither the spleen bacterial loads, nor the serum cytokine levels for the different strains at day 3, were significantly different between the two experiments (results not shown). The cytokines on day 1 and 2 were similar to those found in the uninfected mice and the results are hence not shown. In mice infected with *B*. *suis* 1330, sampled at day 3, 7 and 14, the cytokine levels peaked at day 7 for all cytokines. In mice infected with *B*. *pinnipedialis* 12890 the cytokine levels were similar to those found for *B*. *suis* 1330 but peaked at day 3, except IL-12 (day 7) and IL-5 (low levels at all times, similar to the uninfected mice). *Brucella ceti* elicited a slight increase in IL-12 at day 7 and 14. Otherwise the cytokine levels in mice infected with *B*. *ceti* 12891 were similar to those found in the uninfected mice (Fig 3).

### *In vitro* splenocyte cytokine responses following antigen stimulation

*In vitro* splenocyte cultures from mice 7 days pi were stimulated with the homologous HK *B*. *pinnipedalis* 12890, HK *B*. *ceti* 12891 or HK *B*. *suis* 1330, and 72 h later cell supernatants were evaluated for IFN-γ, TNF-α and IL-6 (S4 Fig). Splenocytes from mice infected with *B*. *pinnipedalis* 12890, *B*. *ceti* 12891 or *B*. *suis* 1330 and stimulated with the homologues HK strain produced significantly more IFN-γ, TNF-α and IL-6 than the splenocytes from mice infected with *B*. *pinnipedalis* 12890, *B*. *ceti* 12891 or *B*. *suis* 1330 stimulated with *E*. *coli* LPS or left unstimulated (S4 Fig).

## Discussion

The pathogenicity of the marine mammal reference strains, *B*. *pinnipedialis* 12890 and *B*. *ceti* 12891 were studied in a BALB/c mouse model. For the recognition of an organism as a member of the genus *Brucella* pathogenicity in mice should be investigated, and the present studies fulfil this requirement [8]. The information from experimental infections in mice is not directly applicable to humans or target animal species and the ideal approach to reveal the infection biology of *Brucella* spp. would be to test the different strains in their natural hosts, but this is impractical and costly, especially when working with strains originating from wild animals, however, by using an appropriate protocol, experiments may yield useful information, allowing comparison of strains [26].

The sole previous experimental infection with a marine mammal *Brucella* spp. in BALB/c mice was with a *B*. *pinnipedialis* hooded seal strain [24]. Molecular studies have characterized the hooded seal *B*. *pinnipedialis* as diverging from other pinniped brucellae, however, a similar lack of *Brucella*-associated pathology [10] and an age-dependent serological and bacteriological pattern, with a low probability of being positive for pups, a higher probability for yearlings, followed by a decreasing probability with age, is identified in both hooded and harbour seals [32, 33, 34]. Yet, when comparing spleen weight in BALB/c mice following experimental infection, there was a discrepancy between the two *Brucella* strains; *B*. *pinnipedialis* hooded seal strain induced no alteration in spleen weight [24], while *B*. *pinnipedialis* 12890 induced an increase in spleen weight at day 7–21. Moreover, when comparing spleen CFU in the same model, there was a difference between *B*. *pinnipedialis* hooded seal strain [24] and *B*. *pinnipedialis* 12890 at day 7, while the numbers thereafter were comparable. Based on a similar behaviour in their host species [32, 33, 34], previous experimental results for *B*. *pinnipedialis* hooded seal strain in BALB/c mice [24] and with the aim of keeping animal experiments to a minimum [35], *B*. *pinnipedialis* 12890 was studied only until day 35 in the present study.

The marine mammal reference strains, *B*. *pinnipedialis* 12890 and *B*. *ceti* 12891, presented attenuated spleen and liver bacterial counts in a BALB/c mouse model of infection and the attenuation was furthermore indicated by the lesser increase in spleen and liver weight, as compared to *B*. *suis* 1330. To our knowledge, the only other wildtype strain attenuated in the mouse model is *B*. *neotomae*, which is considered as not being virulent in its rodent host and having a limited zoonotic potential [36]. This is comparable to the absence of, or limited and unspecific, pathology reported in harbour seals and harbour porpoises infected with *B*. *pinnipedialis* and *B*. *ceti* and the lack of reports of zoonotic incidents [10] among people handling or consuming marine mammals [37].

During the acute phase, extending from day 3 to the time when the CFU reach their highest, brucellae are reported to be readily isolated from blood [26], as shown in the *B*. *suis* 1330 infected mice in the present study. Bacteraemic episodes during the chronic steady phase, however, are said to be transient and scarce [26], yet *B*. *suis* 1330 was isolated from the blood at all times until at least day 56, but the levels after day 21 were low. The longer lasting bacteraemia in the *B*. *ceti* 12891-infected mice than in those infected with *B*. *pinnipedialis* 12890 was in coherence with the lower level and quicker elimination of infection in the *B*. *pinnipedialis* 12890-infected mice, as compared to those infected with *B*. *ceti* 12891.

The bacteriological presence of brucellae in the mouse brain, detected through direct culture, has not been investigated previously, however, the observed behaviour of *Brucella*-infected mice during experimental infections [26], including in the present study, does not suggest brain pathology, as has been documented in humans [38], ungulate foetuses [39] and dolphins [11] where neurobrucellosis is a common clinical trait. Even though the mouse model might not be the appropriate animal model for studying neurobrucellosis and the information from experimental infections in mice is not directly applicable to target animal species [26], the occurrence of neurobrucellosis in dolphins infected with *B*. *ceti* [11] was the rationale for investigating brain tissue in the present study. *Brucella suis* 1330 and *B*. *ceti* 12891 were isolated from brain tissue at times pi when the number of bacteria in the blood was at its highest. Studies point to a relationship between the level of bacteraemia and brain infection for certain pathogens, however, a high bacteraemia level is necessary, but not sufficient, for haematoencephalic barrier adhesion and traversal [40]. The number of bacteria isolated from the brains was maximum 1.61 log CFU/g brain, corresponding to 40.74 CFU/g brain. As the mouse brain is approximately 0.4 g [41] this accounts for 16.30 CFU/brain, which would be a very low level of infection. Moreover, we did not detect any brain pathology in the three brain sections studied per mice and contamination of the brains with blood during sampling cannot be excluded. Yet, mice infected with *B*. *pinnipedialis* 12890 had bacteria in the blood at certain times pi and *B*. *pinnipedialis* 12890 was not isolated from the brains. The use of bioluminescent strains and hence higher ip dosages [42], complemented with direct culture from the organs, could in future provide further knowledge regarding dissemination and tissue localization of marine mammal brucellae in mouse models.

Brucellae were isolated from faecal samples of mice infected with *B*. *suis* 1330 and *B*. *ceti* 12891. Faecal excretion of brucellae has been shown previously in mice following oral infection [43], however, to our knowledge, faecal excretion following ip inoculation has not been described. The isolation of brucellae from faeces coincided with the times when the mice had bacteraemia. Contamination with blood during sampling cannot be excluded, as the faeces were extracted from the large intestine by a mechanical procedure that could have damaged the intestinal blood vessels. Cattle have been shown to shed *B*. *abortus* in faeces [44] and *B*. *pinnipedialis* has been isolated from Pacific harbour seal faeces [32]. The transmission of marine mammal brucellae is still unknown, but the recent publication of a multiplex real-time PCR assay for the detection of brucellae in faeces from marine mammals [45] may shed some light on the role of faecal excretion in the marine mammal brucellae epidemiology.

A detectable increase in the levels of cytokines in serum is indicative of an inflammatory pathogenic process [46]. Most of the lesions observed during brucellosis are a result, either directly or indirectly, of the inflammatory phenomena elicited by *Brucella* spp. [47]. *Brucella suis* 1330 and *B*. *pinnipedialis* 12890 induced similar levels of serum cytokines and both strains produced spleen and liver pathology, indicating that the observed production of cytokines induced tissue damage. The lesions in the *B*. *suis* 1330-infected mice were similar to those described previously for the virulent strains *B*. *abortus* 2308 in BALB/c mice [48] and *B*. *microti* in C57BL/6 mice [49]. The spleen and liver lesions were most pronounced in mice infected with *B*. *suis* 1330, while the pathological changes in mice infected with *B*. *pinnipedialis* 12890 were moderate and transient, similar to those described previously for attenuated brucellae in BALB/c mice [26]. *Brucella ceti* 12891 induced less severe spleen and liver pathology, in fewer mice, than *B*. *pinnipedialis* 12890 and the onset of liver pathology was later, indicating a less pronounced inflammatory response towards *B*. *ceti* as compared to that towards *B*. *pinnipedialis* 12890. To our knowledge this is the first description of spleen and liver histopathology in *B*. *suis* 1330-infected BALB/c mice, as well as in BALB/c mice infected with the marine mammal reference strains.

Defence against microbes is mediated by the early innate immunity and the later responses of the adaptive immunity and there is a delay of 4–7 days before the adaptive immune response takes effect [50]. GM-CSF, IL-1β, TNF-α, IFN-γ, IL-2 and IL-6 are important pro-inflammatory cytokines, part of the nonspecific innate immune response that serves as the first line of defence [46]. The current study showed peaks in serum levels of these cytokines at day 7 pi in the *B*. *suis* 1330-infected mice, as previously described in BALB/c mice infected with *B*. *abortus* 2006018 isolated from a Chinese patient [51] and *B*. *abortus* 2308 [26], whilst in the *B*. *pinnipedialis* 12890-infected mice these cytokines peaked at day 3 pi. Furthermore, when splenocyte cultures from infected mice were stimulated with the homologous HK *Brucella* and the cell supernatants were evaluated for IFN-γ, TNF-α and IL-6, both *B*. *suis* 1330 and *B*. *pinnipedialis* 12890 produced significantly more cytokines than the splenocytes from infected mice stimulated with *E*. *coli* LPS or left unstimulated, confirming that both strains elicit a release of these cytokines in response to specific stimulation also *in vitro*. The results presented here suggest that *B*. *pinnipedalis* 12890 might lack some of the virulence factors present in *B*. *suis* 1330 which allow the latter to escape the immune response and establish a chronic infection more efficiently [2].

IL-12 bridges innate resistance and adaptive immunity [52] and primes the Th1 immune response characteristic for adaptive immunity against *Brucella* spp. [25]. IL-12/β2-microglobulin knockout mice infected with *B*. *abortus* 2308 have shown increased numbers of bacterial load in spleens as compared to C57BL/6 mice [53] and administration of anti-IL-12 monoclonal antibodies to *B*. *abortus* 19 infected CBA mice led to an exacerbation of the infection [54]. Previous studies have shown a peak in serum levels of IL-12 in BALB/c mice infected with *B*. *abortus* 2308 at around 7 days pi [25] and the same pattern was observed for both *B*. *suis* 1330 and *B*. *pinnipedialis* 12890 in the current study indicating a priming of the Th1 immune response in mice infected with both strains. Indeed, the cytokine patterns *in vivo* for the Th2 cytokines IL-4, IL-5 and IL-10 followed a similar pattern for *B*. *suis* 1330 and *B*. *pinnipedialis* 12890 in the current study, with not significantly different levels at day 3, 7 and 14 pi.

The earlier cytokine response in the *B*. *pinnipedialis* 12890-infected mice is followed by a reduced level of cytokines at day 7 pi as compared to *B*. *suis* 1330. This is a time pi when the adaptive immunity has taken effect [50] and *Brucella* spp. typically express Th1-cytokines, as shown for *B*. *suis* 1330 in the present study. However, one may speculate whether the prompt inflammatory reaction towards *B*. *pinnipedialis* 12890, as shown by the cytokine profiles *in vivo* and *in vitro*, the earlier peak in spleen weight and the inflammatory responses in the spleen and liver, may have been efficient to a level where a Th1 response is induced to a lesser extent. Experimental infections with *B*. *pinnipedialis* 12890 in mice with mutations in genes coding for key factors of the innate and adaptive immune response could provide further insight into the properties of the immune response towards *B*. *pinnipedialis* 12890 in the mouse model.

The prompt immune response and the clearance of infection in the BALB/c mouse model, however, is comparable to what is seen in harbour seals, where age-dependent serological and bacteriological patterns for *B*. *pinnipedialis* have been identified, with a low probability of being positive for pups, a higher probability for yearlings, followed by a decreasing probability with age, suggesting clearance of infection [32, 33]. The same pattern has also been identified in hooded seals [34], hence, *B*. *pinnipedialis* could be causing an acute transient infection in pinnipeds, which could also explain the limited gross pathology [10] found in pinnipedis infected with *B*. *pinnipedialis*.

The serum cytokine levels in mice infected with *B*. *ceti* 12891 were similar to those found in the uninfected mice. However, when splenocyte cultures from *B*. *ceti* 12891-infected mice were stimulated with the homologous HK *Brucella* and the cell supernatants were evaluated for IFN-γ, TNF-α and IL-6, splenocytes produced significantly more cytokines than the splenocytes from infected mice stimulated with *E*. *coli* LPS or left unstimulated, confirming that the BALB/c mouse immune system is able to mount a specific immune response against *B*. *ceti* 12891, as well as *B*. *pinnipedialis* 12890 and *B*. *suis* 1330. The antibody isotype levels also confirm the presence of an immune response against all three strains of brucellae. The immunological pattern described for *B*. *ceti* 12891, in combination with persistence in the organs with limited signs of pathology, has heretofore not been seen for any other species of brucellae and further investigations are needed to draw any conclusions regarding the immunological response in the BALB/c mouse model to *B*. *ceti* 12891. The predominantly similar antibody isotype patterns between the virulent *B*. *suis* 1330 and the attenuated marine mammal reference strains was as previously described for virulent and attenuated strains of brucellae [31].

In dolphins, *B*. *ceti* has been isolated from lesions in the reproductive systems, and from aborted foetuses, vaginal secretions, milk and mammary glands, and in addition, several times from the CNS associated with neurological pathology. Further investigations should be made in the mouse model with *B*. *ceti* dolphin strains, as pathology has been described in the natural host [11]. In contrast, there are some reports of pathology in porpoises in association to infection with *B*. *ceti*, but the number of incidences are fewer, in general less severe and with unspecific pathological signs [11, 55]. The attenuation of *B*. *ceti* 12891 porpoise strain in the present study is thus in line with the restricted pathogenicity in porpoises. However, there are significant discrepancies in both innate and adaptive immunity mechanisms between species [56] and a transfer of the herein observed properties to porpoises should be made with caution.

There are approximately 35 living species of pinnipeds distributed all over the world [57] and isolations of *B*. *pinnipedialis* have been made in seven of these [10, 12]. The cetaceans are a large group with approximately 78 species [58] and *B*. *ceti* has been isolated from thirteen cetacean species; [10, 12, 59]. Considering that brucellae have been isolated from approximately 20% of the pinniped and cetacean species, while anti-*Brucella* antibodies have been detected in several more of the species [10–12], one should assume that we still have an “Ocean of *Brucella*” to explore, and that new information related to zoonotic potential, bacteriology, serology, genetics, host species, host-pathogen-interaction, and transmission are probably yet to come.

## Supporting Information

S1 Fig Liver bacterial counts and organ weights. Liver bacterial counts for B. *pinnipedialis* 12890 (open diamonds), *B*. *ceti* 12891 (crosses) and *B*. *suis* 1330 (black squares) in livers (a) of BALB/c mice after intraperitoneal (ip) inoculation of 10^5^ colony forming units (CFU) of bacteria. Uninfected control mice received sterile phosphate buffered saline ip (black lines). Four or five mice were euthanized per lot at day 3, 7, 14, 21, 35, 56 and 84 post infection (day 56 and 84; only *B*. *ceti* 12891 and *B*. *suis* 1330). The number of viable bacteria was determined, and the numbers of bacterial counts were logarithmic transformed. Liver weights (b) were determined in parallel. Results are expressed as mean + one standard deviation. Whether CFU numbers differed significantly between mice infected with *B*. *suis* 1330, and *B*. *ceti* 12891 or *B*. *pinnipedialis* 12890, respectively, at the different times pi, are presented in Table 1. Whether liver weights of the infected mice were significantly different from those of the uninfected control mice, at the different times pi, is presented in S1 Table.
(TIF)

S2 Fig Blood and faeces bacterial counts. Presence of *B*. *pinnipedialis* 12890 (open diamonds), *B*. *ceti* 12891 (crosses) and *B*. *suis* 1330 (black squares) per ml of blood (a) and per gram of faeces (b) in BALB/c mice after intraperitoneal inoculation of 10^5^ colony forming units (CFU) of bacteria. Uninfected control mice received sterile phosphate buffered saline ip. Four or five mice were euthanized per lot at day 3, 7, 14, 21, 35, 56 and 84 post infection (day 56 and 84; only *B*. *ceti* 12891 and *B*. *suis* 1330). The number of viable bacteria was determined. The numbers of bacteria in the blood were logarithmic transformed, while the numbers of bacteria in the faeces are presented as CFU/gram. Results are expressed as mean + one standard deviation. Whether results differed significantly between mice infected with *B*. *suis* 1330, or *B*. *ceti* 12891 and *B*. *pinnipedialis* 12890, respectively, at the different times pi, are presented in Table 1.
(TIF)

S3 Fig Spleen and liver histopathology at day 14 post infection. Spleen and liver histopathology in BALB/c mice after intraperitoneal (ip) inoculation of 10^5^ of *B*. *suis* 1330, *B*. *pinnipedialis* 12890 or *B*. *ceti* 12891. Uninfected control mice received sterile phosphate buffered saline ip. Spleens (2x): a, b, c and d, (20x): e, f, g and h. Mice infected with *B*. *suis* 1330 had mildly affected spleen architecture with small and ill-defined lymphoid follicles (lymphoid depletion), no germinal centers and an expanded red pulp (a). A 20x enlargement of an ill-defined lymphoid follicle with scant numbers of lymphocytes from a *B*. *suis* 1330 infected mouse is presented (e). Mice infected with *B*. *pinnipedialis* 12890 had preserved spleen architecture with small lymphoid follicles, some of them with germinal centers (b and f). The spleens of mice infected with *B*. *ceti* 12891 had preserved architecture with well-demarcated lymphoid follicles, some of them with germinal centers (c). A 20x enlargement of a lymphoid follicle with the marginal zone present from a *B*. *ceti* 12891-infected mouse is presented (g). Uninfected mouse spleens with no lesions (2x and 20x, d and h). Livers (20x): i, j, k and l. Mice infected with *B*. *suis* 1330 showing multiple well-defined inflammatory nodules in the liver characterized by macrophages and neutrophils, with some of the nodules extending and coalescing with each other (i). Mice infected with *B*. *pinnipedialis* 12890 (j) and *B*. *ceti* 12891 (k) showing small well-demarcated granulomas scattered throughout the liver tissue. Uninfected mouse livers with no lesions (20x, l).
(TIFF)

S4 Fig Splenocyte supernatant cytokines. Level of interferon (IFN)-γ (a), tumor necrosis factor (TNF)-α (b) and interleukin (IL) -6 (c) in splenocyte supernatants from BALB/c mice infected by intraperitoneal (ip) inoculation of 10^5^ colony forming units of *B*. *pinnipedialis* 12890 (dark grey), *B*. *ceti* 12891 (black)or *B*. *suis* 1330 (light grey) 7 days earlier. Uninfected mice received sterile phosphate buffered saline ip (white). Splenocytes were either stimulated with the homologous HK *B*. *pinnipedalis* 12890, HK *B*. *ceti* 12891 or HK *B*. *suis* 1330, or left unstimulated (medium). Additionally, splenocytes from uninfected mice were stimulated with the same HK brucellae, or left unstimulated. As controls, splenocytes from infected and uninfected mice were stimulated with LPS from *Escherichia coli*. The experiments were repeated twice. The results are presented as mean + one standard error of the mean. *** = p < 0.001, ** = p < 0.01, * = p < 0.05.
(TIFF)

S1 Table Level of statistical difference between organ weights in uninfected and infected mice. Level of statistical difference between spleen and liver weights in uninfected and infected BALB/c mice after intraperitoneal (ip) inoculation of 10^5^ colony forming units (CFU) of *B*. *suis* 1330, B. *pinnipedialis* 12890 or *B*. *ceti* 12891. Uninfected mice received sterile phosphate buffered saline ip. Four or five mice were euthanized per lot at day 3, 7, 14, 21, 35, 56 and 84 post infection (day 56 and 84; only *B*. *ceti* 12891 and *B*. *suis* 1330). Infected mice were compared to uninfected mice and *** = p < 0.001, ** = p < 0.01, * = p < 0.05, ns = not significant, X = not available.
(DOCX)
