# Supplementary figures and images for: Marine Mammal Brucella Reference Strains Are Attenuated in a BALB/c Mouse Model
Source: PLoS One. 2016 Mar 9;11(3):e0150432. doi: 10.1371/journal.pone.0150432 (PMC4784796; doi:10.1371/journal.pone.0150432)

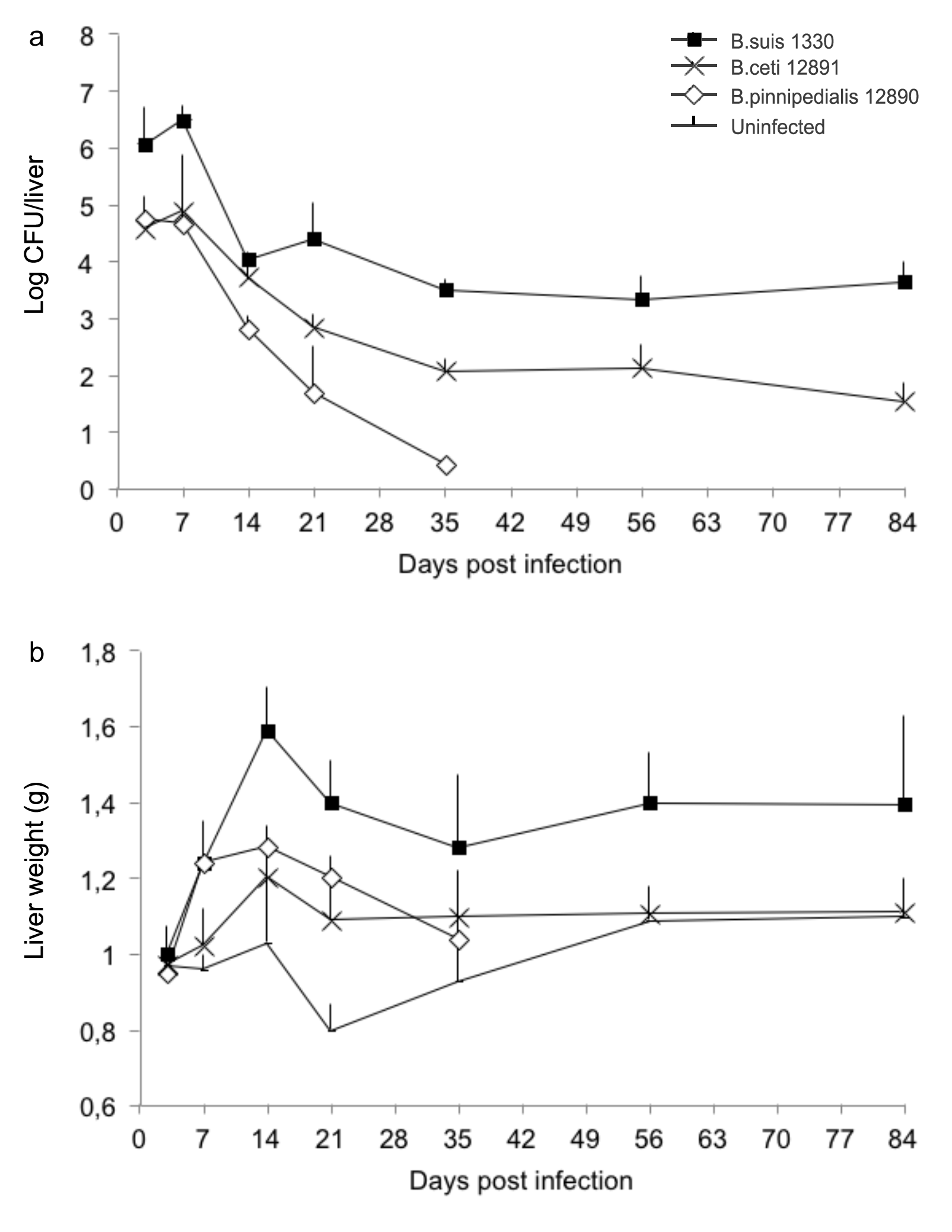

Supplement: S1 Fig — Liver bacterial counts for B. pinnipedialis 12890 (open diamonds), B. ceti 12891 (crosses) and B. suis 1330 (black squares) in livers (a) of BALB/c mice after intraperitoneal (ip) inoculation of 105 colony forming units (CFU) of bacteria. Uninfected control mice received sterile phosphate buffered saline ip (black lines). Four or five mice were euthanized per lot at day 3, 7, 14, 21, 35, 56 and 84 post infection (day 56 and 84; only B. ceti 12891 and B. suis 1330). The number of viable bacteria was determined, and the numbers of bacterial counts were logarithmic transformed. Liver weights (b) were determined in parallel. Results are expressed as mean + one standard deviation. Whether CFU numbers differed significantly between mice infected with B. suis 1330, and B. ceti 12891 or B. pinnipedialis 12890, respectively, at the different times pi, are presented in Table 1. Whether liver weights of the infected mice were significantly different from those of the uninfected control mice, at the different times pi, is presented in S1 Table. (TIF) [file pone.0150432.s001.tif]

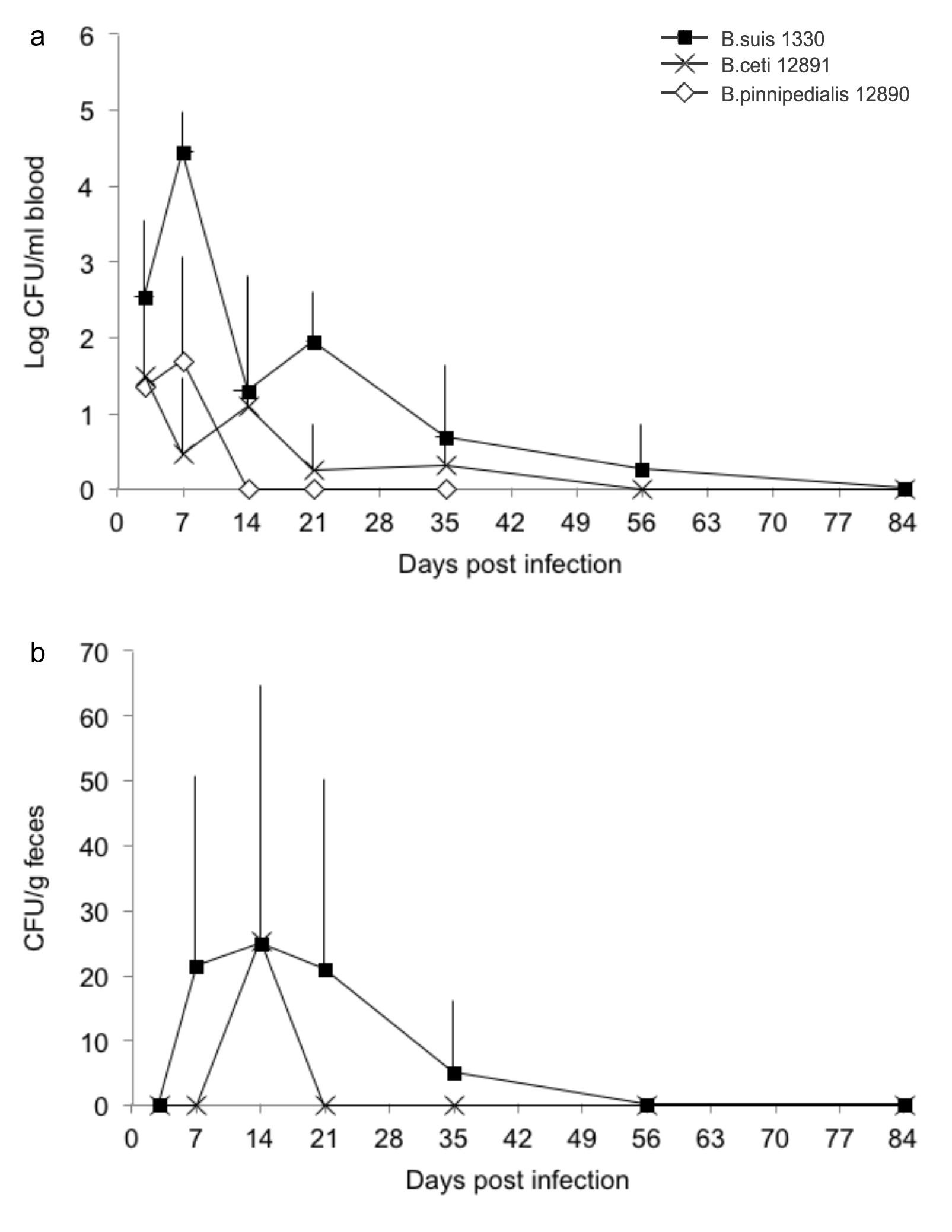

Supplement: S2 Fig — Presence of B. pinnipedialis 12890 (open diamonds), B. ceti 12891 (crosses) and B. suis 1330 (black squares) per ml of blood (a) and per gram of faeces (b) in BALB/c mice after intraperitoneal inoculation of 105 colony forming units (CFU) of bacteria. Uninfected control mice received sterile phosphate buffered saline ip. Four or five mice were euthanized per lot at day 3, 7, 14, 21, 35, 56 and 84 post infection (day 56 and 84; only B. ceti 12891 and B. suis 1330). The number of viable bacteria was determined. The numbers of bacteria in the blood were logarithmic transformed, while the numbers of bacteria in the faeces are presented as CFU/gram. Results are expressed as mean + one standard deviation. Whether results differed significantly between mice infected with B. suis 1330, or B. ceti 12891 and B. pinnipedialis 12890, respectively, at the different times pi, are presented in Table 1. (TIF) [file pone.0150432.s002.tif]

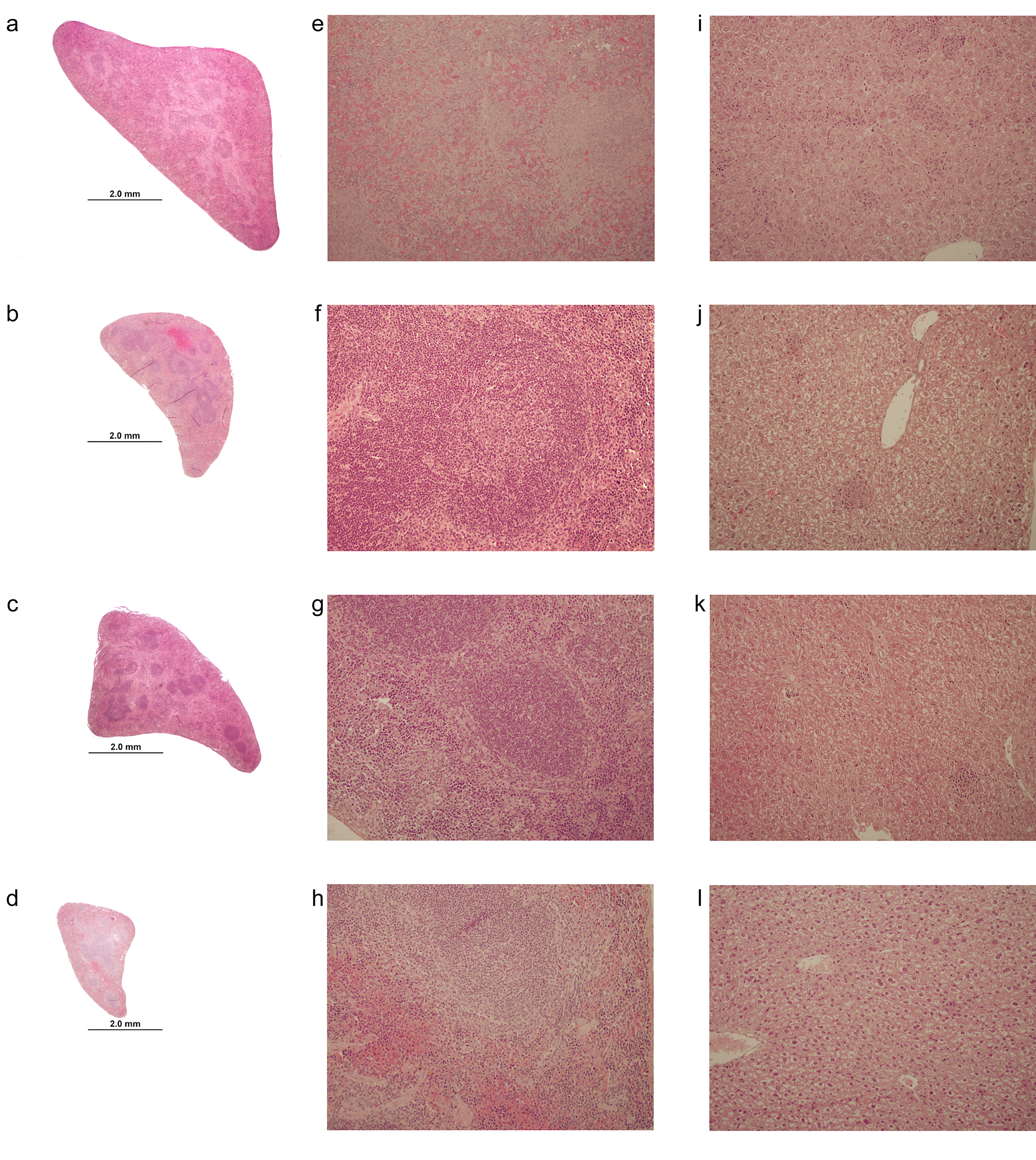

Supplement: S3 Fig — Spleen and liver histopathology in BALB/c mice after intraperitoneal (ip) inoculation of 105 of B. suis 1330, B. pinnipedialis 12890 or B. ceti 12891. Uninfected control mice received sterile phosphate buffered saline ip. Spleens (2x): a, b, c and d, (20x): e, f, g and h. Mice infected with B. suis 1330 had mildly affected spleen architecture with small and ill-defined lymphoid follicles (lymphoid depletion), no germinal centers and an expanded red pulp (a). A 20x enlargement of an ill-defined lymphoid follicle with scant numbers of lymphocytes from a B. suis 1330 infected mouse is presented (e). Mice infected with B. pinnipedialis 12890 had preserved spleen architecture with small lymphoid follicles, some of them with germinal centers (b and f). The spleens of mice infected with B. ceti 12891 had preserved architecture with well-demarcated lymphoid follicles, some of them with germinal centers (c). A 20x enlargement of a lymphoid follicle with the marginal zone present from a B. ceti 12891-infected mouse is presented (g). Uninfected mouse spleens with no lesions (2x and 20x, d and h). Livers (20x): i, j, k and l. Mice infected with B. suis 1330 showing multiple well-defined inflammatory nodules in the liver characterized by macrophages and neutrophils, with some of the nodules extending and coalescing with each other (i). Mice infected with B. pinnipedialis 12890 (j) and B. ceti 12891 (k) showing small well-demarcated granulomas scattered throughout the liver tissue. Uninfected mouse livers with no lesions (20x, l). (TIFF) [file pone.0150432.s003.tiff]

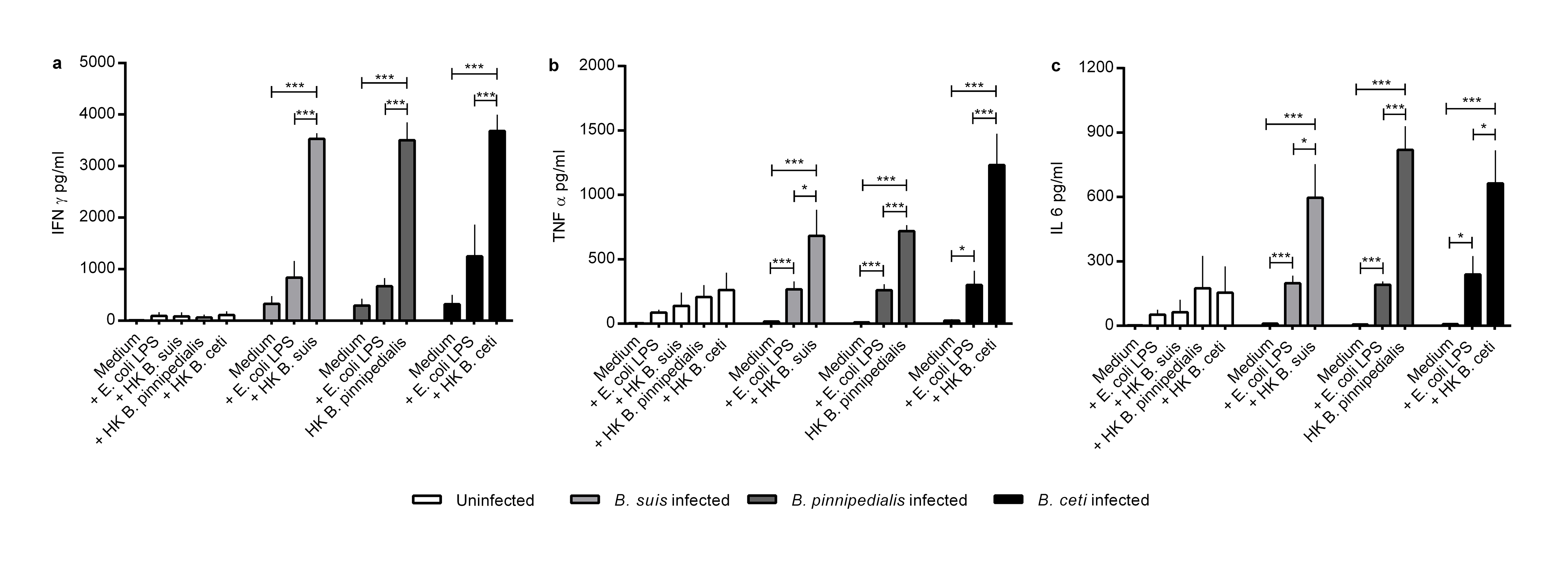

Supplement: S4 Fig — Level of interferon (IFN)-γ (a), tumor necrosis factor (TNF)-α (b) and interleukin (IL) -6 (c) in splenocyte supernatants from BALB/c mice infected by intraperitoneal (ip) inoculation of 105 colony forming units of B. pinnipedialis 12890 (dark grey), B. ceti 12891 (black)or B. suis 1330 (light grey) 7 days earlier. Uninfected mice received sterile phosphate buffered saline ip (white). Splenocytes were either stimulated with the homologous HK B. pinnipedalis 12890, HK B. ceti 12891 or HK B. suis 1330, or left unstimulated (medium). Additionally, splenocytes from uninfected mice were stimulated with the same HK brucellae, or left unstimulated. As controls, splenocytes from infected and uninfected mice were stimulated with LPS from Escherichia coli. The experiments were repeated twice. The results are presented as mean + one standard error of the mean. *** = p < 0.001, ** = p < 0.01, * = p < 0.05. (TIFF) [file pone.0150432.s004.tiff]
