# Supplementary material for: Marine Mammal Brucella Reference Strains Are Attenuated in a BALB/c Mouse Model
Source: PLoS One. 2016 Mar 9;11(3):e0150432. doi: 10.1371/journal.pone.0150432 (PMC4784796; doi:10.1371/journal.pone.0150432)
Supplement: S1 Table — Level of statistical difference between spleen and liver weights in uninfected and infected BALB/c mice after intraperitoneal (ip) inoculation of 105 colony forming units (CFU) of B. suis 1330, B. pinnipedialis 12890 or B. ceti 12891. Uninfected mice received sterile phosphate buffered saline ip. Four or five mice were euthanized per lot at day 3, 7, 14, 21, 35, 56 and 84 post infection (day 56 and 84; only B. ceti 12891 and B. suis 1330). Infected mice were compared to uninfected mice and *** = p < 0.001, ** = p < 0.01, * = p < 0.05, ns = not significant, X = not available. (DOCX) [file pone.0150432.s005.docx]

**S1 Table. Level of statistical difference between organ weights in uninfected and infected mice.**

| Day | Strain | 3 | 7 | 14 | 21 | 35 | 56 | 84 |
| --- | --- | --- | --- | --- | --- | --- | --- | --- |
| **Spleen weight** | *Brucella suis* 1330 | * | *** | *** | ** | ** | ** | ** |
|  | *Brucella pinnipedialis* 12890 | ns | ns | ** | ** | * | X | X |
|  | *Brucella ceti* 12891 | ns | *** | ** | ** | ns | * | ns |
| **Liver weight** | *Brucella suis* 1330 | ns | ** | ** | *** | * | * | ns |
|  | *Brucella pinnipedialis* 12890 | ns | ns | ns | ** | * | X | X |
|  | *Brucella ceti* 12891 | ns | *** | * | *** | ns | ns | ns |
